# Negative pressure wound therapy for the prevention of surgical site infections in orthopedic and trauma surgery: a systematic review and meta-analysis of RCTs

**DOI:** 10.1186/s10195-025-00889-0

**Published:** 2025-12-16

**Authors:** Ibrahim Ebeid, Ahmed Ebeid, Ahmed Shalaby, Ibrahim Mohamed Noureldeen, Ali Essa, Khaled A. Elmenawi

**Affiliations:** 1 Faculty of Medicine, Menoufia University, Shebeen El-Kom, Menoufia Egypt; 2 Department of Orthopaedic Surgery, Cleveland Clinic, Cleveland, OH USA

**Keywords:** Orthopedics, Surgical site infections (SSIs), Negative pressure wound therapy (NPWT), Meta-analysis

## Abstract

**Background:**

Surgical site infections (SSIs) are an important postoperative complication in orthopedic surgery, resulting in increased morbidity, prolonged hospital stay, and higher healthcare costs. Negative pressure wound therapy (NPWT) has been proposed to reduce SSIs by facilitating wound healing by increased perfusion, edema reduction, and bacterial control. This systematic review and meta-analysis evaluate the effectiveness of NPWT compared with conventional dressings for prevention of surgical site infections in orthopedic and trauma surgery.

**Methods:**

A comprehensive literature search was performed across PubMed, Web of Science, Scopus, and the Cochrane Library in December 2024. Only randomized controlled trials (RCTs) comparing NPWT with CD in patients undergoing joint replacement, trauma surgery, or spine surgery were included. Two independent reviewers conducted data extraction and assessed study quality using the Cochrane Risk of Bias 2 tool. Pooled outcomes were evaluated with odds ratios (ORs) computed for dichotomous variables and mean differences (MDs) for continuous outcomes. Heterogeneity was assessed via the I^2^ statistic and publication bias through Egger’s test.

**Results:**

Overall, 18 RCTs, comprising a total of 4585 patients, were included. Meta-analysis demonstrated that NPWT significantly reduced SSIs (pooled OR 0.64, 95% CI 0.50–0.82; *p* = 0.0005) and wound dehiscence (pooled OR 0.39, 95% CI 0.23–0.65; *p* = 0.0003). Additionally, NPWT was associated with a reduction in length of hospital stay by 0.87 days (MD −0.87, 95% CI −1.36 to −0.38; *p* = 0.0005) and fewer dressing changes compared with conventional methods. The quality of evidence for the primary outcome was rated as moderate based on the GRADE approach.

**Conclusions:**

NPWT appears to offer a significant clinical benefit in reducing the incidence of SSIs in orthopedic and trauma surgery. Secondary analyses also demonstrated benefits for surgical wound dehiscence, length of hospital stay, and number of dressing changes. However, the certainty of evidence is moderate, and these findings should be interpreted with caution. Further well-designed, multicenter RCTs are warranted to confirm these benefits, assess long-term outcomes, and evaluate cost-effectiveness.

*Level of evidence* Level I.

*Registration:* CRD42024624188.

**Supplementary Information:**

The online version contains supplementary material available at 10.1186/s10195-025-00889-0.

## Introduction

Surgical site infections (SSIs) are a major challenge in post-operative care, contributing significantly to morbidity, mortality, and healthcare costs. SSIs refer to infections that occur within 30 days after surgery or up to one year after implant placement [1]. SSIs occur in 2.5%–11% of surgical patients, with orthopedic procedures being particularly vulnerable due to the use of prosthetic materials and microbial colonization of bone and soft tissues [2–5]. Among orthopedic surgeries, SSI prevalence varies between 0.91% and about 18% in high-risk cases such as open fractures or revision surgeries [6–12]. SSIs significantly increase healthcare costs. In large US cohorts, each SSI was associated with approximately 8–9 extra hospital days and $19,000–$21,000 in additional costs [13]. In orthopedic surgeries, the financial burden is even higher. Postoperative infections after total hip or knee arthroplasty increased costs by approximately $20,000–$40,000 for superficial SSIs and $50,000–$75,000 for deep infections [14, 15]. Even in smaller orthopedic cohorts, such as knee allograft procedures, patients with confirmed or suspected deep infections incurred an average of $12,100 in added healthcare costs [16]. Beyond financial costs, SSIs reduce the quality of life through chronic pain, functional limitations, and impaired mobility, while increasing mortality risk by 2- to 11-fold [17, 18].

Negative pressure wound therapy (NPWT) has emerged as an effective strategy for reducing the risk of surgical site infections (SSIs). While NPWT is the most widely used term in literature, some authors have noted that it is a misnomer, as true negative pressure is a physical impossibility [19]. Nevertheless, we use the term NPWT in this article owing to its broad acceptance in both clinical practice and scientific literature. It furthers wound healing by enhancing the synthesis of crucial growth factors, such as vascular endothelial growth factor (VEGF) and fibroblast growth factor 2 (FGF-2), while simultaneously reducing the expression of proinflammatory cytokines [20]. NPWT also helps decrease the bacterial burden in wounds and maintains a moist environment, thereby creating a more favorable environment for tissue repair [21, 22].

Advanced NPWT systems, including NPWT with instillation and dwell time (NPWTi-d), improve wound care by performing wound bed cleansing and contaminant dilution and solubilization while removing infectious material debris and exudate to promote granulation tissue formation [23, 24]. Additionally, newer modalities like incisional NPWT (iNPWT)—a sealed device applied over closed surgical incisions have demonstrated promise in reducing SSIs among high-risk patients [25, 26]. However, conflicting evidence persists: While studies are showing that NPWT lowers the rates of infection compared with standard dressings [27, 28], some studies have shown no statistically significant difference [29–31].

Several factors increase the risk of SSIs, including high BMI (> 35), longer operative time (> 120 min), excessive bleeding, and simultaneous bilateral procedures are significant risk factors [32]. Comorbidities like diabetes, kidney disease, liver disease, and vascular issues also raise risk [33]. General anesthesia increases infection risk, while tranexamic acid offers protection [34]. Certain bacteria, such as S. aureus and Gram-negative organisms, are more common in early SSIs [35]. Additionally, recent evidence highlights that the surgical site itself is an independent risk factor for SSIs in orthopedic procedures, with trauma surgeries showing a higher likelihood of infection compared with spine or joint surgeries [36]. International guidelines differ in their recommendations: the National Institute for Health and Care Excellence (NICE) supports NPWT for high-risk incisions, whereas the World Health Organization (WHO) supports conventional dressings due to cost considerations [37, 38].

While SSIs are the most prevalent postoperative morbidity, surgical wound dehiscence (SWD) is another critical postoperative complication that may precede infection. SWD occurs in 1%–3% of orthopedic procedures and often presents with or precedes incisional SSIs, owing to overlapping risk factors such as poor tissue oxygenation, malnutrition, and mechanical tension on the incision [39–41]. SWD typically occurs between postoperative days 5 and 8 and reflects impaired wound healing [42]. Negative pressure wound therapy (NPWT) has been shown to reduce the risk of SWD by improving tissue perfusion, minimizing edema, and stabilizing wound edges [43]. However, current evidence remains sparse, with few randomized clinical trials (RCTs) evaluating SWD as a standalone outcome [27, 44]. This systematic review and meta-analysis aim to synthesize data from RCTs to clarify the effectiveness of NPWT in preventing surgical site infections in orthopedic and trauma surgery; secondary outcomes on wound dehiscence, hospital stay, and dressing changes are also reported.

## Methods

### Study design

The systematic review and meta-analysis were conducted in accordance with the Preferred Reporting Items for Systematic Reviews and Meta-Analyses (PRISMA) guidelines. The primary objective was to evaluate the effectiveness of NPWT in preventing postoperative wound complications following orthopedic surgeries. The review protocol was prospectively registered with PROSPERO (CRD42024624188). To ensure a high-quality evidence base, the study focused exclusively on RCTs. The inclusion criteria were carefully defined: studies must be RCTs involving patients who underwent orthopedic and trauma surgery, comparing the application of NPWT as a primary wound management strategy against conventional wound dressings. Studies were only included if they reported on at least one of the following outcomes: the primary outcome of surgical site infections (SSI) (superficial or deep), or the secondary outcomes of surgical wound dehiscence (SWD), length of hospital stay, or the number of dressing changes. Conversely, a rigorous exclusion process was applied to filter out non-RCT designs such as case reports, case series, cohorts, and nonrandomized trials, as well as conference abstracts and animal studies. Studies involving nonorthopedic surgeries or those not specifically comparing NPWT with conventional dressings were also excluded. Furthermore, any studies lacking data on the specified outcomes or duplicate publications were removed to maintain the integrity of the review. For feasibility and to ensure consistency of reporting, we restricted our systematic search to full-text RCTs published in English and indexed in PubMed, Scopus, Web of Science, and the Cochrane Library. We did not search for grey literature, nonEnglish publications, or unpublished data in trial registries.

### Search strategy

A comprehensive search strategy was developed and applied to retrieve relevant studies in August 2025. The following databases were searched: PubMed, Web of Science, Scopus, and Cochrane Library. The search was conducted using a combination of MeSH terms, keywords, and Boolean operators. Key search terms included “negative pressure wound therapy,” “orthopedic surgery”, and “surgical wound infection.” Full details of the search strategy, including search strings and database-specific adaptations, are provided in supplementary material.

### Study selection

All identified articles were imported to Rayyan [45] for reference management, and duplicates were removed. Two independent reviewers (A.E. and A.S.) screened titles and abstracts against the inclusion criteria. Full-text screening was then performed to confirm eligibility. Discrepancies were resolved through discussion or by consulting a third reviewer (I.E.).

### Data Extraction

Data were extracted independently by two reviewers (A.E. and I.N.) using a standardized data extraction form. Extracted data were mainly divided into 3 domains: (1) summary of study characteristics, (2) characteristics of the included studies’ population, and (3) study outcomes. Any discrepancies in data extraction were resolved by consensus or consultation with a third reviewer (I.E.).

### Risk of bias assessment

We assessed the risk of bias of each included study using the Cochrane Risk of Bias 2 (RoB 2) tool. The following domains were evaluated: (1) randomization process, (2) deviations from intended interventions, (3) missing outcome data, (4) measurement of the outcome, and (5) selection of the reported result. Each domain was rated as “low risk,” “some concerns,” or “high risk” of bias.

### Publication bias

Publication bias was assessed using Egger’s regression test [46], which evaluates funnel plot asymmetry by regressing standardized effect estimates against their precision. A statistically significant intercept (*p* < 0.05) indicates potential publication bias. The results were complemented by visual inspection of a funnel plot.

### Data synthesis and statistical analysis

Data synthesis and meta-analysis were carried out using RevMan 5.4 software [47]. For dichotomous outcomes (SSIs and SWD), the results were expressed as odds ratios (OR) with 95% confidence intervals (CIs). For continuous outcomes (Length of hospital stay, number of dressing changes), mean differences (MD) with 95% CIs were calculated. For studies or outcomes with missing standard deviations (SDs), we estimated SDs using reported summary statistics when possible. Specifically, SDs were calculated from reported p-values of t-tests or from CIs of means or mean differences using standard formulas. When only medians and interquartile ranges (IQRs) were available, these were converted to means and SDs following the method described by Wan et al. methodology [48]. Details of estimations are in supplementary material. The meta-analysis was conducted using both fixed and random effects models, depending on the degree of heterogeneity observed across studies.

### Assessment of heterogeneity

Heterogeneity was estimated using the Chi-square test and I^2^ statistic. Significant heterogeneity was defined as an I^2^ value > 50% or a p-value < 0.10. To investigate potential sources of heterogeneity, sensitivity analyses and subgroup analyses were performed based on factors such as the type of surgery. In cases of significant heterogeneity, a random-effects model was employed to account for the variability across studies. If no significant heterogeneity was detected, a fixed-effects model was used. The overall assessment of heterogeneity helped guide the interpretation of the pooled estimates and the robustness of the findings.

### GRADE assessment

The certainty of the evidence for each outcome was evaluated using the Grading of Recommendations, Assessment, Development, and Evaluations (GRADE) approach. The quality of evidence was rated as “high,” “moderate,” “low,” or “very low” on the basis of the following factors: risk of bias, inconsistency, indirectness, imprecision, and publication bias. GRADE evidence profiles were created for each outcome to provide a transparent summary of the strength of evidence.

### Deviations from the protocol

Our review was conducted in accordance with the a priori protocol registered with PROSPERO (CRD42024624188). However, during the review process, the following minor deviations from the original protocol were implemented to enhance the quality and relevance of our findings.

First, the original search date was specified as December 2024. To ensure the inclusion of the most current evidence, we updated our systematic literature search to include studies published through August 2025. Second, while the original protocol focused on orthopedic surgeries, we broadened the scope to include “trauma surgery” in the final review title and related sections of the manuscript. Finally, we performed a sensitivity analysis excluding high-risk-of-bias studies and were unable to perform funnel plots or Egger’s test for certain outcomes due to having fewer than 10 studies, in accordance with the Cochrane Handbook for Systematic Reviews of Interventions. All other aspects of the review, including the eligibility criteria, risk of bias assessment using the RoB 2 tool, and data synthesis plan, were followed as outlined in the registered protocol.

### Ethical considerations

Since this study is a review of previously published data, ethical approval was not required. However, ethical principles for systematic reviews, such as transparency, integrity, and rigorous adherence to PRISMA guidelines, were followed throughout the review process.

## Results

### Literature search results

**Fig. 1:**
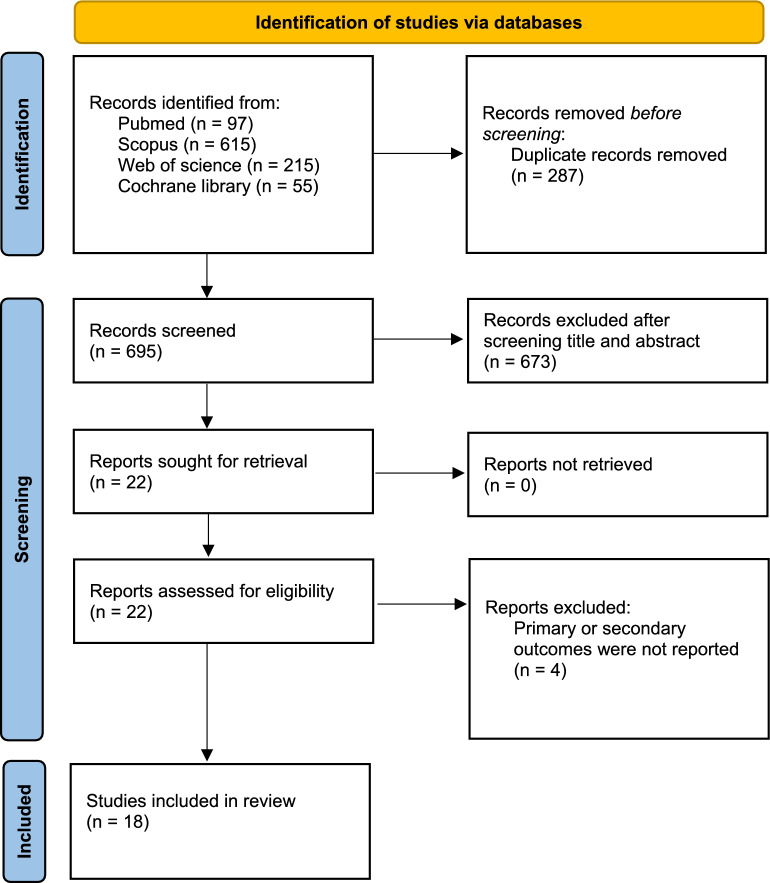
PRISMA flow diagram of the study selection process

A total of 928 articles were identified through database searches. Of them, 264 were identified as duplicate articles by Rayyan. During the title and abstract screening, 642 articles were excluded for not meeting the inclusion criteria. The full texts of 22 articles were assessed for eligibility, with 4 articles excluded following a detailed review. Ultimately, 18 studies were included in the quantitative synthesis (meta-analysis). The PRISMA flow diagram of the study selection process is shown in Fig. 1.

### Characteristics of included studies

The analysis included a total of 18 randomized controlled trials (RCTs) [27–31, 44, 49–60], encompassing 8 studies on trauma surgery, 9 on joint surgeries (hip/knee arthroplasty), and 1 on spinal surgery. Overall, the research included 4585 patients, 2270 going through NPWT, and 2315 receiving conventional dressings. Elaborate descriptions of the studies, including population characteristics, surgical procedures, and key findings, are provided in Table 1. Population demographics, such as age, BMI, gender distribution, and sample sizes for NPWT and conventional.

**Table 1:** Summary of included studies

Study	Country	Population	Type of surgery	Findings
Arti et al. (2016) [49]	Iran	Patients aged 15–55 years with Gustilo-Anderson type IIIB open fracture wounds and clean wounds after debridement	Open fracture wound management	NPWT significantly reduced wound healing duration and hospitalization days in comparison to conventional dressings No significant difference in infection rates between groups
Cai et al. (2022) [50]	China	Patients with fresh closed unilateral calcaneal fractures less than two weeks old, a normal contralateral calcaneus without prior issues, and a minimum follow-up of 12 months	Open reduction and internal fixation (ORIF)	The vacuum sealing drainage (VSD) group showed significantly better outcomes in terms of faster wound healing, less pain, and fewer complications compared with conventional drainage
Canton et al. (2020) [51]	Italy	Patients undergoing open reduction and internal fixation (ORIF) for ankle (bi- or tri-malleolar) and distal tibia fractures who had risk factors such as age > 65 years, smoking, BMI > 30 (obesity), or diabetes	ORIF for ankle and distal tibial fractures	iNPWT demonstrated a trend toward fewer minor wound complications compared with conventional dressing (6.3% versus 30.6%; *p* = 0.091), while major complications were comparable between groups (6.3% versus 4.1%), with no significant differences observed in overall complication rates and no device-related adverse effects reported
Cooper et al. (2022) [52]	USA	High-risk patients undergoing direct anterior total hip arthroplasty (DA THA), defined as those with a BMI > 30 kg/m^2^, diabetes mellitus, active smoking, or a history of prior open hip surgery	THA	Surgical site complications (SSCs) occurred in 18.3% of the control group compared with 8.3% in the closed incision negative pressure therapy (ciNPT) group, though this difference was not statistically significant (*p* = 0.107). However, superficial SSIs were significantly lower in the ciNPT group (3.3% versus 15.0%; *p* = 0.027)
Costa et al. (2018) [30]	UK	Adults aged ≥ 16 years with an open lower limb fracture (Gustilo and Anderson grades 2 or 3) presenting within 72 h of injury	Open fracture management of lower limbs involving surgical debridement, followed by internal or external fixation	No significant difference in disability rating index (DRI) scores at 12 months (mean difference: −3.9 points, *p* = 0.132) Similar rates of surgical site infections and complications NPWT did not improve clinical or economic outcomes
Costa et al. (2020) [31]	UK	Patients aged ≥ 16 years with a lower limb fracture caused by major trauma requiring surgery, and a wound that could be closed	Surgery for lower limb fractures associated with major trauma	No significant difference in deep SSI rates at 30 days (5.84% in the NPWT group versus 6.68% in the standard dressing group) No significant differences in secondary outcomes
Crist et al. (2017) [29]	USA	Patients aged 18 years or older undergoing ORIF for acetabular fractures	ORIF for acetabular fractures	No statistically significant reduction in deep infections with NPWT (6.1% in gauze versus 15.2% in NPWT, *p* = 0.25) ICU stay significantly longer for infected patients (7.3 versus 2.3 days, *p* < 0.05)
Giannini et al. (2018) [53]	Italy	40–80-year-old patients undergoing hip or knee prosthetic revision for aseptic loosening	Hip or knee revision surgery	ASEPSIS scores were significantly lower in the NPWT group (mean: 3.0) versus control (mean: 5.1) (*p* < 0.001), but not clinically relevant except for high-risk patients NPWT reduced dressing changes and pain scores significantly compared with controls
Gillespie et al. (2015) [54]	Australia	Adults attending the preadmission clinic who were able to provide informed consent	Primary THA	Dressing costs were significantly lower in the control group ($3.01/day versus $38.40/day). The incidence of SSIs was 2/35 in the NPWT group and 3/35 in the control group, with an intention-to-treat risk ratio (RR) of 0.67 (95% CI: 0.12–3.7; *p* = 0.65). However, NPWT patients had significantly more postoperative wound complications (RR = 1.6; 95% CI: 1.0–2.5; *p* = 0.04)
Higuera-Rueda et al. (2021) [44]	North America	Patients aged ≥ 22 years undergoing revision total knee arthroplasty (TKA) for aseptic or septic reasons, or ORIF of periprosthetic fractures, classified as high-risk for surgical site complications (SSCs) based on criteria such as BMI > 35 kg/m^2^, diabetes, or tobacco use	Revision TKA and ORIF of periprosthetic fractures	ciNPT group had significantly lower 90-day SSCs (3.4% versus 14.3%; OR 0.22; *p* = 0.0013), readmissions (3.4% versus 10.2%; *p* = 0.0208), and dressing changes (mean 1.1 versus 1.3; *p* = 0.0003)
Karlakki et al. (2016) [55]	UK	Patients undergoing elective primary THA or primary TKA	Primary THA and Primary TKA	Significant reduction in dressing changes in the intervention group (mean difference: 1.7, *p* = 0.002) Four-fold reduction in wound complications in the intervention group (8.4% control versus 2.0%, *p* = 0.06) Length of hospital stay reduced by 0.9 days but not statistically significant (*p* = 0.07)
Keeney et al. (2019) [56]	USA	Patients undergoing primary or revision hip and knee total joint arthroplasty	Primary and revision THA and TKA	iNPWT was associated with improved short-term wound healing but not long-term infection rates Significant benefits observed for patients with BMI > 35 kg/m^2^ undergoing TKA, with reduced complications and dressing concerns No difference in late superficial or deep infection rates
Manoharan et al. (2016) [57]	Australia	Patients undergoing primary knee arthroplasty	Primary knee arthroplasty	NPWT reduced wound leakage and improved wound protection but had no significant benefit in wound healing or economic costs compared with conventional dry dressings
Masters et al. (2021) [58]	UK	Patients aged ≥ 65 years undergoing surgery for hip fractures	Various hip fracture surgeries including hemiarthroplasty, total hip replacement (THR), and internal fixation	At 30 days, the rate of deep SSIs was significantly lower in the iNPWT group at 1.9%, compared with 6.4% in the standard dressing group. This corresponds to a risk ratio (RR) of 0.29 (95% CI 0.10–0.85), indicating a substantial reduction in the risk of deep SSIs with NPWT. The overall mortality rate at 30 days across both groups was 7.6%
Newman et al. (2019) [59]	USA	Patients undergoing revision THA or TKA who were at high risk of infection	Revision THA and revision TKA	Significant reduction in total wound complications in the intervention group (10.1% versus 23.8%, *p* = 0.022) Reoperation rates lower in the intervention group (2.5% versus 12.5%, *p* = 0.017)
Pérez-Acevedo et al. (2024) [60]	Spain	Pediatric patients (< 18 years) with nonidiopathic scoliosis (NIS) undergoing surgery, with legal guardian consent required	Spinal deformity correction	Significant reduction in the incidence of SSCs with the use of iNPWT (7.7% vs 38.5% in the control group (per-protocol analysis)) Both groups exhibited comparable median healing times of 7 days iNPWT proved to be cost-effective, substantially reducing costs associated with complications compared with conventional care
Stannard et al. (2012) [27]	USA	Patients older than 18 years with high-energy tibial plateau, pilon, or calcaneus fractures	ORIF for high-risk lower extremity fractures	NPWT significantly reduced total infections (10% in NPWT group versus 19% in control group, *p* = 0.049) SWD was lower in the NPWT group (8.6%) compared with the control group (16.5%, *p* = 0.044)

*NPWT* negative pressure wound therapy; *iNPWT* incisional negative pressure wound therapy; *ciNPT* closed incision negative pressure therapy; *VSD* vacuum sealing drainage; *ORIF* open reduction and internal fixation; *THA* total hip arthroplasty; *TKA* total knee arthroplasty; *DRI* disability rating index; *BMI* body mass index; *SSC* surgical site complication; *SSI* surgical site infection; *SWD* surgical wound dehiscence; *THR* total hip replacement; *NIS* nonidiopathic scoliosis; *RCT* randomized controlled trial; *h* hours

Dressing groups across studies, are outlined in Table 2.

**Table 2:** Summary of the characteristics of the population in the included studies

Study ID	Age				BMI				Sex (females only)		Sample Size	
	NPWT		CD		NPWT		CD		NPWT	CD	NPWT	CD
	Mean	SD	Mean	SD	Mean	SD	Mean	SD				
Arti et al. (2016)	31.86	9.7	31.86	9.7	NA	NA	NA	NA	11	11	45	45
Cai et al. (2022)	43.1	9.2	40.4	10.7	NA	NA	NA	NA	17	21	55	53
Canton et al. (2020)	62.53	15.85	66.33	11.46	27.67	6.1	26	4.58	10	33	16	49
Cooper et al. (2022)	63.6	NA	64.4	NA	32.5	NA	32.9	NA	38	37	60	60
Costa et al. (2018)	44	23.88	42	23.12	NA	NA	NA	NA	48	70	226	234
Costa et al. (2020)	NA	NA	NA	NA	26.4	5.9	26.7	6	302	281	785	763
Crist et al. (2017)	44.2	NA	43.2	NA	29.9	NA	31	NA	6	13	33	33
Giannini et al. (2018)	66	8.9	66.8	11.5	27.7	4.3	28.2	4.7	31	32	50	50
Gillespie et al. (2015)	63.8	14	62.5	12.4	29.9	5.7	29.8	5.3	15	18	35	35
Higuera-Rueda et al. (2021)	64.7	9.48	65.1	8.51	34.7	6.73	34.2	7.18	83	92	147	147
Karlakki et al. (2016)	69	9	69.2	9	30.1	5	28.4	4.6	53	52	102	107
Keeney et al. (2019)	60.6	NA	60.5	NA	34.6	NA	36.5	NA	112	123	185	213
Manoharan et al. (2016)	NA	NA	NA	NA	NA	NA	NA	NA			21	21
Masters et al. (2021)	84.07	9.7	83.63	8.95	NA	NA	NA	NA	160	168	232	230
Newman et al. (2019)	65	11	65	11	31.9	7.5	33.4	7.5	39	35	79	80
Pérez-Acevedo et al. (2024)	11.88	3.21	12	4.79	18.6	5.06	20.08	5.63	12	5	26	26
Stannard et al. (2012)	43	NA	43	NA	NA	NA	NA	NA	46	42	130	119
Virani et al. (2016)	34.8	NA	37.4	NA	NA	NA	NA	NA	15	18	43	50

*NPWT* negative pressure wound therapy, *CD* conventional dressing, *SD* standard deviation, *NA* not applicable

### Risk of bias

**Fig. 2:**
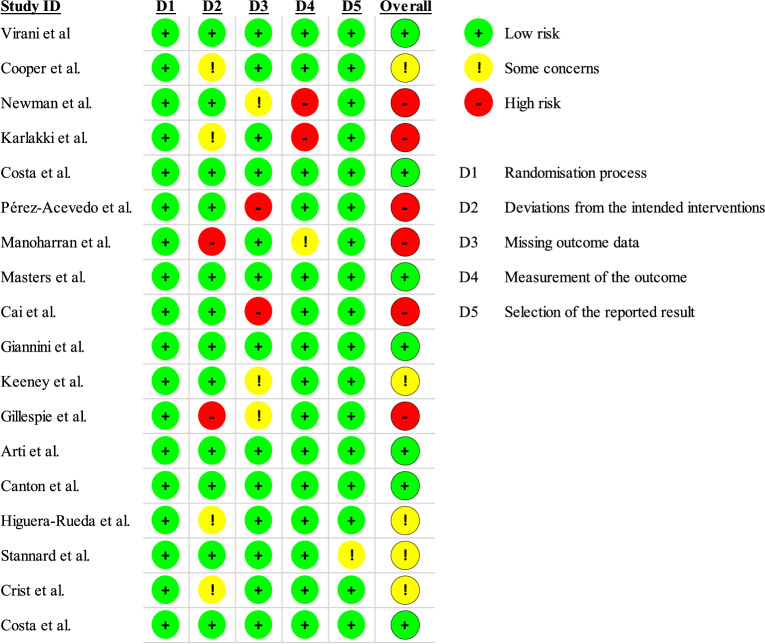
Risk of bias assessment of the included RCTs

All 18 RCTs were assessed for risk of bias using the RoB 2 tool [51]. Out of 18 trials, seven were rated as low risk, five as having some concerns, and six as high risk. All studies had low risk for the randomization process, but deviations from intended interventions were a frequent issue. Missing outcome data was high risk in two studies and raised some concerns in three others. Outcome measurement was mostly low risk, except for three studies. Selective reporting was generally low risk, with only one study rated as low risk. A detailed breakdown of all RoB 2 domains for each study is presented in Fig. 2 and supplementary material.

### Surgical site infections

**Fig. 3:**
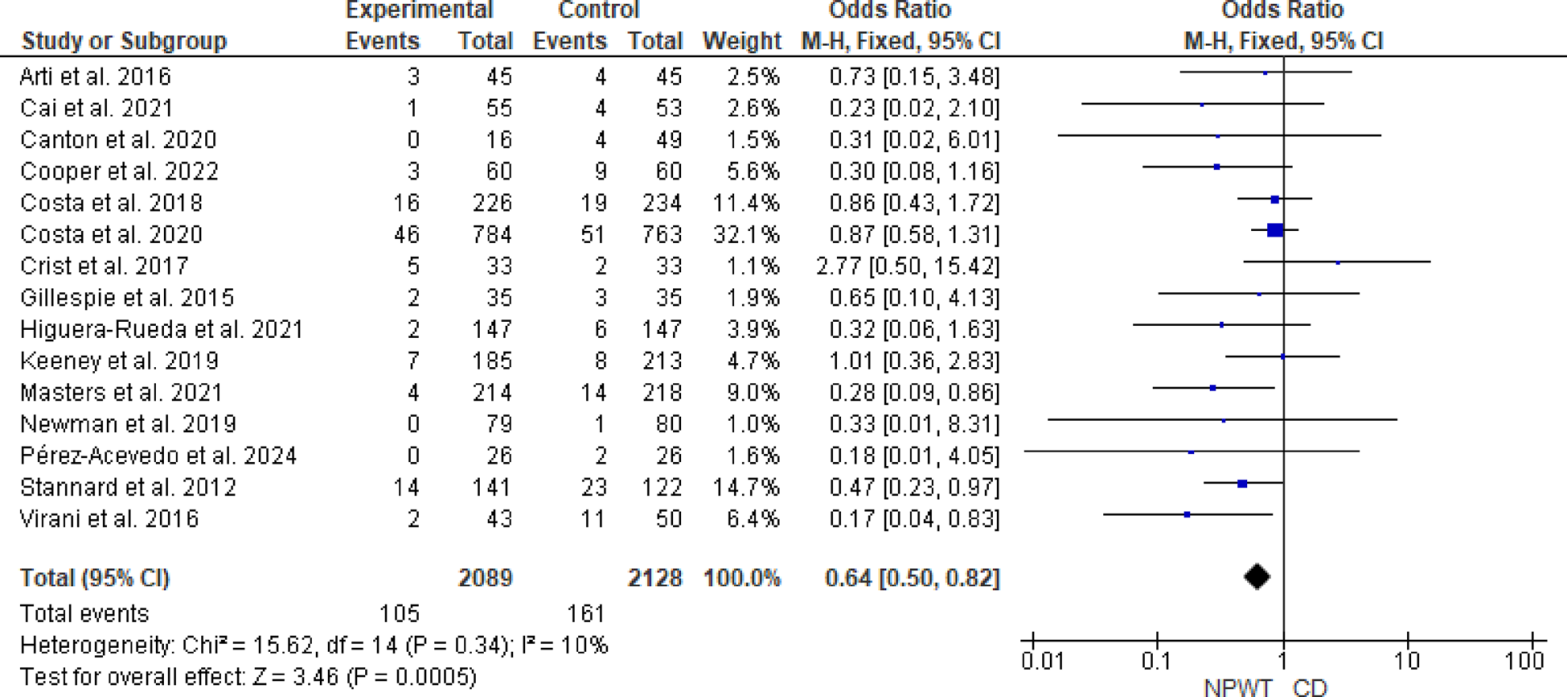
Forest plot of the meta-analysis comparing NPWT and CD for the incidence of surgical site infections. Blue squares represent the OR for each included study, with the size of the square proportional to the study’s weight in the fixed-effects meta-analysis. Horizontal lines indicate 95% confidence CI and the black diamond represents the pooled OR. *NPWT* negative pressure wound therapy; *CD* conventional dressing; *CI* confidence interval; *OR* odds ratio

The meta-analysis of SSIs demonstrated a significant reduction in odds with NPWT compared with CD, with a pooled OR of 0.64 (95% CI 0.50–0.82, *p* = 0.0005). Heterogeneity among studies was not significant (I^2^ = 10%, *p* = 0.34). Figure 3.

**Fig. 4:**
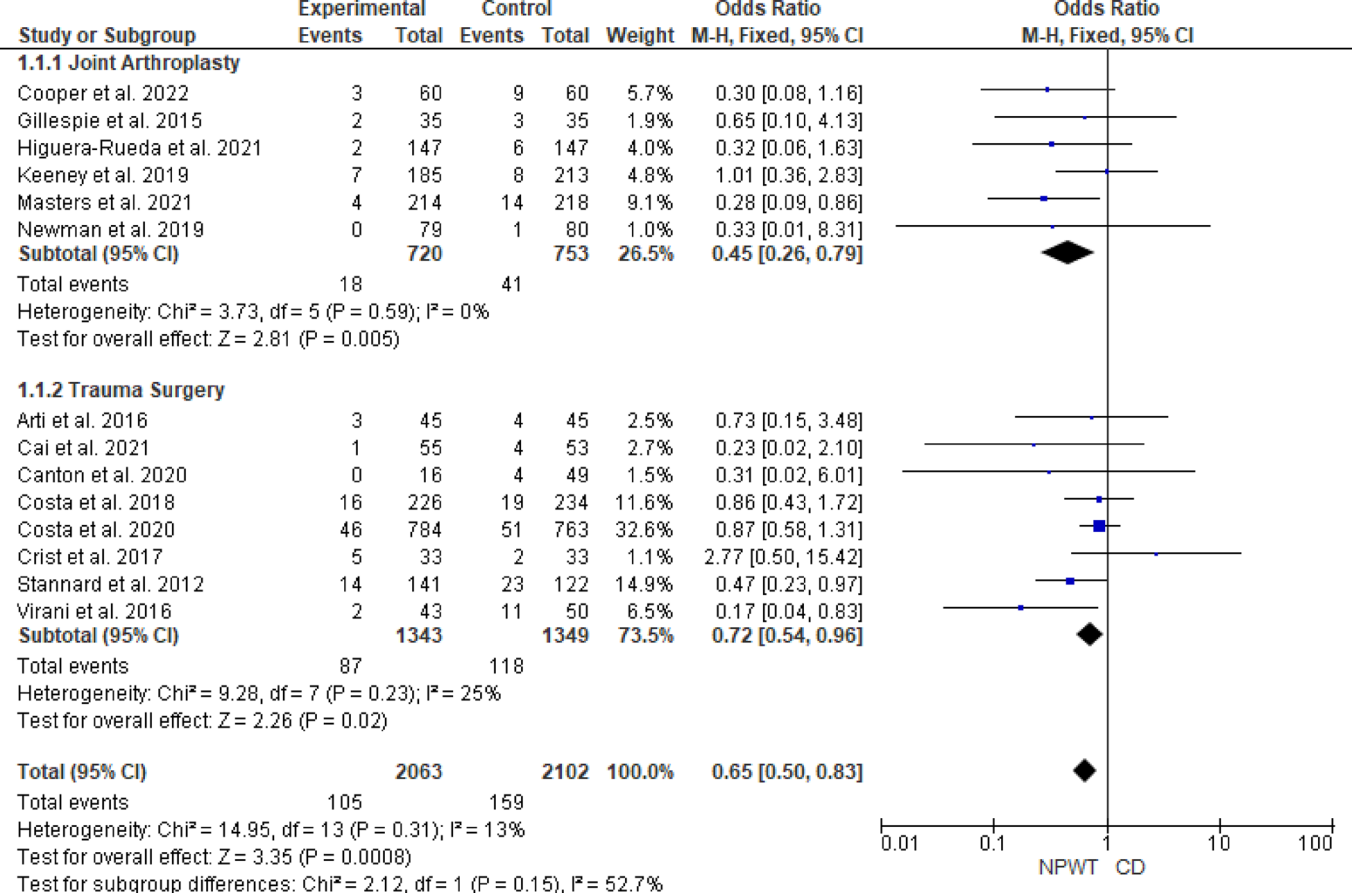
Forest plot of the subgroup analysis comparing the effectiveness of NPWT and CD in reducing surgical site infections across joint arthroplasty and trauma surgery. Blue squares represent the OR for each included study, with the size of the square proportional to the study’s weight in the fixed-effects meta-analysis. Horizontal lines indicate 95% CI and the black diamond represents the pooled OR within each subgroup. *NPWT* negative pressure wound therapy; *CD* conventional dressing; *CI* confidence interval; *OR* odds ratio

The subgroup analysis showed that for joint arthroplasty, the pooled OR was 0.45 (95% CI 0.26–0.79, *p* = 0.005), indicating a statistically significant reduction in SSIs with NPWT compared with CD, with no heterogeneity observed (I^2^ = 0%, *p* = 0.59). Similarly, for trauma surgery, the pooled OR was 0.72 (95% CI 0.54–0.96, *p* = 0.02), also showing a statistically significant reduction in SSIs with NPWT and low heterogeneity (I^2^ = 25%, *p* = 0.23). The subgroup difference test suggested no significant variation between the two surgical subgroups (*p* = 0.15), indicating consistent effectiveness of NPWT across both contexts. Figure 4.

**Fig. 5:**
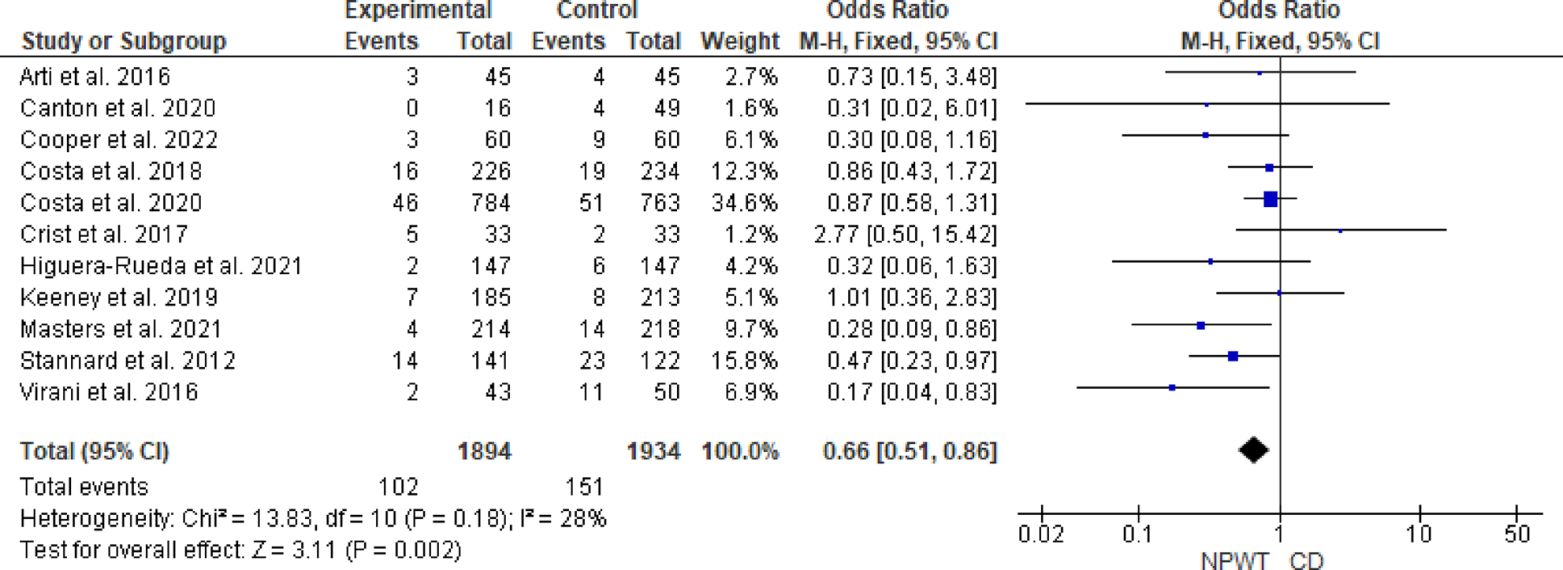
Forest plot of the meta-analysis comparing NPWT and CD for the incidence of surgical site infections after excluding high-risk studies. Blue squares represent the OR for each included study, with the size of the square proportional to the study’s weight in the fixed-effects meta-analysis. Horizontal lines indicate 95% CI and the black diamond represents the pooled OR. *NPWT* negative pressure wound therapy; *CD* conventional dressing; *CI* confidence interval; *OR* odds ratio

After excluding high-risk-of-bias studies, meta-analysis of 11 RCTs demonstrated that NPWT was associated with a statistically significant reduction in the pooled OR of SSIs compared with CD (OR = 0.66, 95% CI 0.51–0.86, *p* = 0.002). Heterogeneity was low (I^2^ = 28%, *p* = 0.18). Figure 5.

### Surgical wound dehiscence

**Fig. 6:**
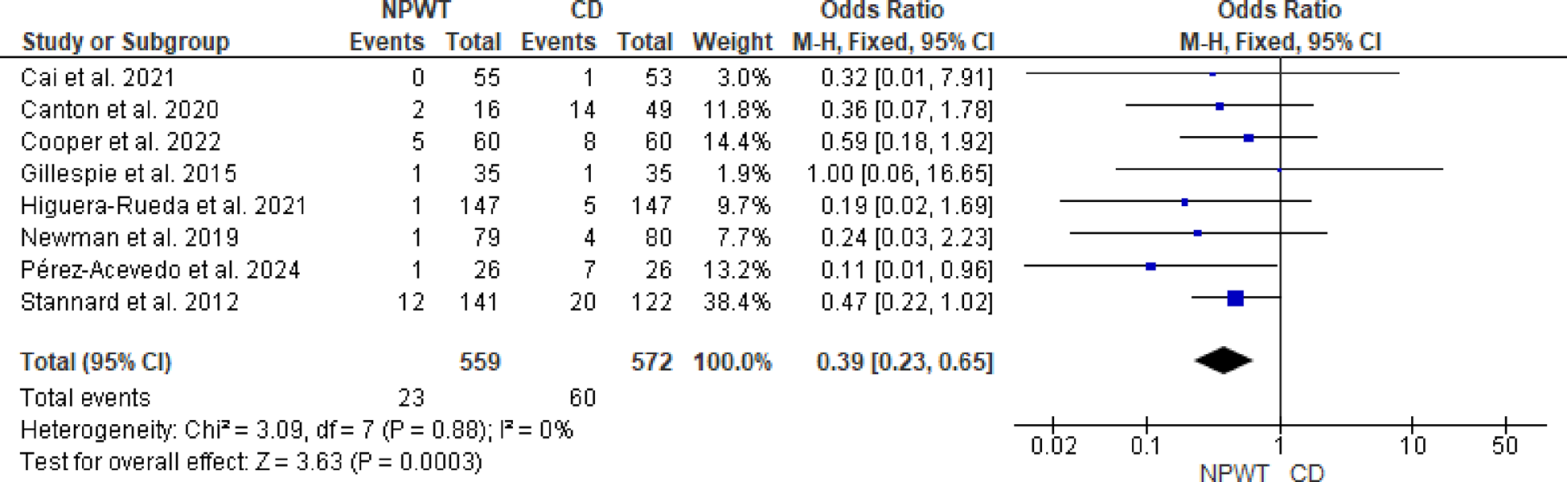
Forest plot of the meta-analysis comparing NPWT and CD for the incidence of surgical wound dehiscence. Blue squares represent the OR for each included study, with the size of the square proportional to the study’s weight in the fixed-effects meta-analysis. Horizontal lines indicate 95% confidence *CI* and the black diamond represents the pooled OR. *NPWT* negative pressure wound therapy; *CD* conventional dressing; *CI* confidence interval; *OR* odds ratio

The overall OR was 0.39 (95% CI 0.23–0.65, *p* = 0.0003), indicating that NPWT significantly reduces the risk of SWD to CD. There was no observed heterogeneity among the studies (I^2^ = 0%, *p* = 0.88). Figure 6.

### Length of hospital stay

**Fig. 7:**
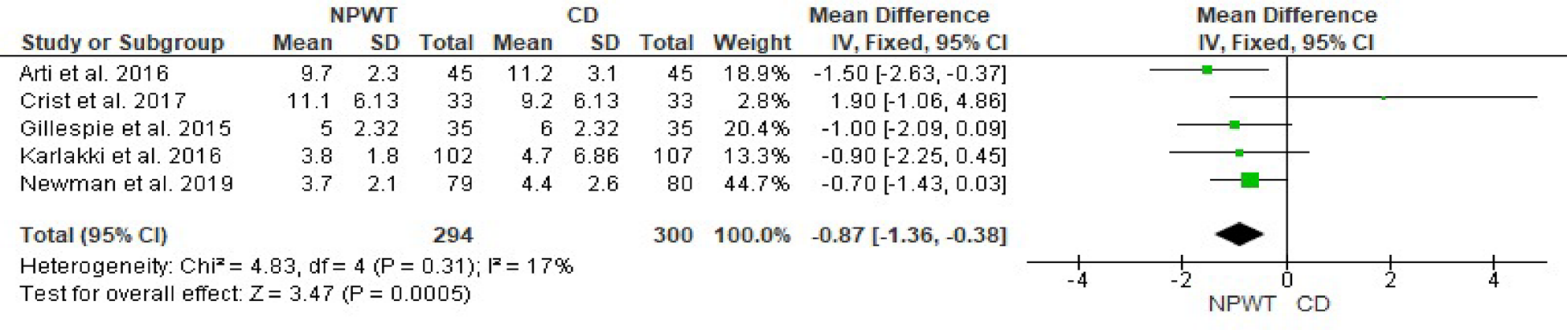
Forest plot comparing the effect of NPWT versus CD on length of hospital stay. Green squares represent the MD for each included study, with the size of the square proportional to the study’s weight in the fixed-effects meta-analysis. Horizontal lines indicate 95% CI, and the black diamond represents the pooled MD. *NPWT* negative pressure wound therapy; *CD* conventional dressing; *CI* confidence interval; *MD* mean difference

The overall mean difference (MD) was −0.87 days (95% CI −1.36 to −0.38, *p* = 0.0005), showing that NPWT is associated with a significantly shorter hospital stay compared with CD. Heterogeneity was not significant (I^2^ = 17%, *p* = 0.31), suggesting consistency in the results across studies. Figure 7.

### Number of dressing changes

**Fig. 8:**
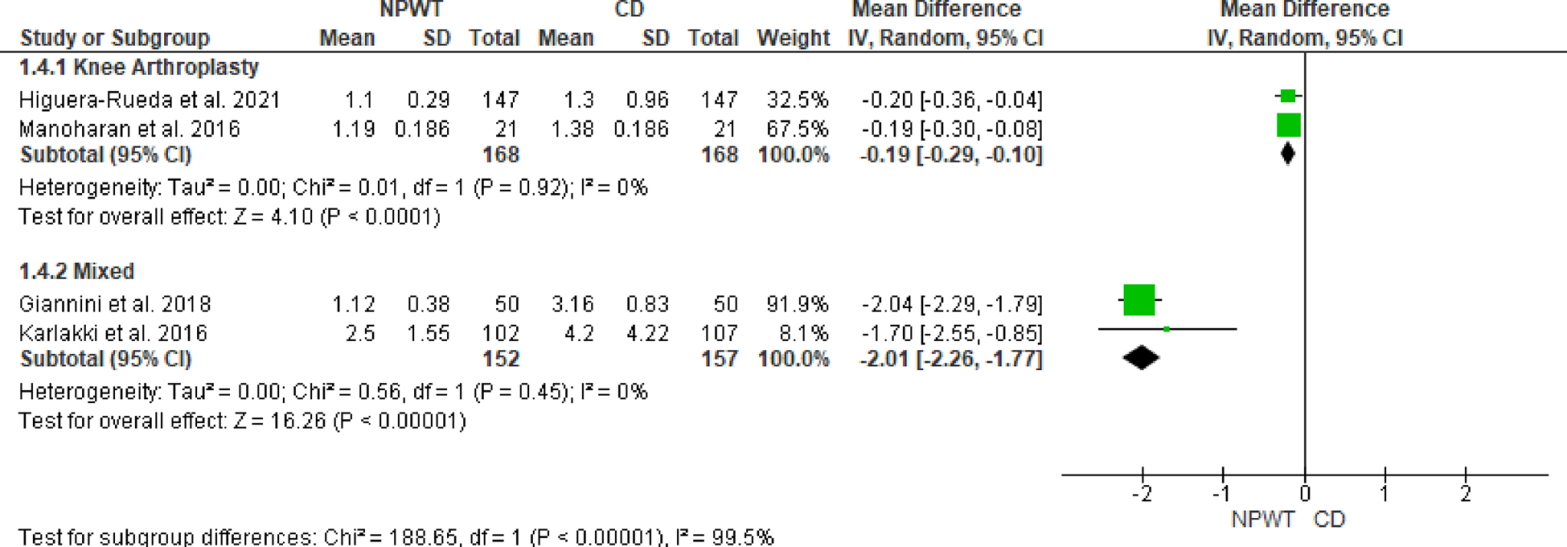
Forest plot of the subgroup analysis comparing the effect of NPWT versus CD on the number of dressing changes in knee arthroplasty and mixed arthroplasty groups. Green squares represent the MD for each included study, with the size of the square proportional to the study’s weight in the random-effects meta-analysis. Horizontal lines indicate 95% CI, and the black diamond represents the pooled MD within each subgroup. *NPWT* negative pressure wound therapy; *CD* conventional dressing; *CI* confidence interval; *MD* mean difference

The subgroup analysis categorized studies into knee arthroplasty and mixed arthroplasty groups; the mixed arthroplasty group included those RCTs in which either hip arthroplasty or knee arthroplasty had been performed, and both groups showed no significant heterogeneity. For knee arthroplasty, the MD was −0.19 (95% CI −0.29 to −0.10), demonstrating a significant reduction in dressing changes with NPWT (*p* < 0.0001). In the mixed arthroplasty group, the MD was −2.01 (95% CI −2.26 to −1.77), also showing a significant reduction with NPWT (*p* < 0.0001). Figure 8.

### Publication bias

**Fig. 9:**
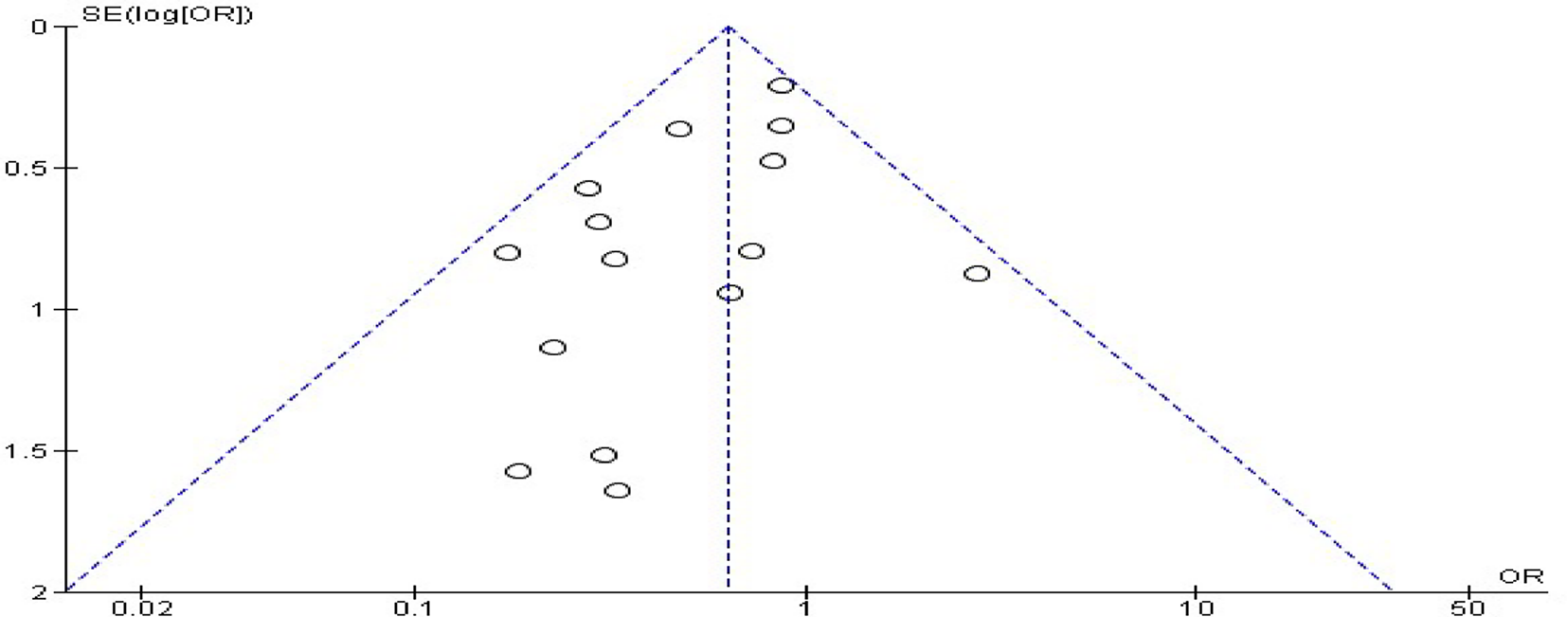
Funnel plot assessing publication bias for studies evaluating the effect of NPWT on surgical site infections. Each dot represents an individual study, plotted according to its OR on the x-axis and SE on the y-axis. NPWT: negative pressure wound therapy; OR: odds ratio; SE: standard error

Egger’s regression test revealed a statistically significant intercept of −0.9389 (*p* = 0.0487), suggesting potential publication bias in the included studies. The funnel plot (Fig. 9) displayed asymmetry, with smaller studies clustered toward the left side of the plot, further supporting the possibility of bias. This asymmetry may reflect underrepresentation of smaller studies with nonsignificant or negative results, potentially leading to overestimation of NPWT’s efficacy.

### GRADE assessment

On the basis of the GRADE assessment (Table 3), the evidence for the reduction in SSIs with NPWT compared with conventional dressings was evaluated as being of moderate quality, downgraded by one level due to potential publication bias, as indicated by the statistically significant Egger’s test (*p* = 0.0487) and asymmetry in the funnel plot. For SWD, the evidence quality was moderate but downgraded due to some studies having a high risk of bias. For the length of hospital stay, the evidence was of low quality because many studies had a high risk of bias. The number of dressing changes showed very low-quality evidence due to high risk of bias and significant heterogeneity among studies.

**Table 3:** GRADE assessment of the certainty in evidence for each outcome

Certainty assessment							Summary of findings				
Participants (studies) Follow-up	Risk of bias	Inconsistency	Indirectness	Imprecision	Publication bias	Overall certainty of evidence	Study event rates (%)		Relative effect (95% CI)	Anticipated absolute effects	
							With CD	With NPWT		Risk with CD	Risk difference with NPWT
SSIs											
4217 (15 RCTs)	Not serious	Not serious	Not serious	Not serious	Publication bias strongly suspected^a^	⨁⨁⨁◯ Moderate^a^	161/2128 (7.6%)	105/2089 (5.0%)	**OR 0.64** (0.50 to 0.82)	161/2128 (7.6%)	**26 fewer per 1,000** (from 36 to 13 fewer)
SWD											
1131 (8 RCTs)	serious^b^	Not serious	Not serious	Not serious	None^c^	⨁⨁⨁◯ Moderate^b^	60/572 (10.5%)	23/559 (4.1%)	**OR 0.39** (0.23 to 0.65)	60/572 (10.5%)	**61 fewer per 1,000** (from 79 to 34 fewer)
Length of Hospital Stay											
594 (5 RCTs)	Very serious^d^	Not serious	Not serious	Not serious	None^c^	⨁⨁◯◯ Low^d^	300	294	–	300	MD **0.87 days fewer** (1.36 fewer to 0.38 fewer)
Number of dressing changes											
309 (4 RCTs)	Very serious^e^	Serious^f^	Not serious	Not serious	None^c^	⨁◯◯◯ Very low^e,f^	157	152	-	157	MD **2.01 fewer** (2.26 fewer to 1.77 fewer)

*CI* confidence interval, *MD* mean difference, *OR* odds ratio; *CD* conventional dressing, *NPWT* negative pressure wound therapy, *SSIs* surgical site infections, *SWD* surgical wound dehiscence; *RCT* randomized controlled trial

*Explanations*

a. Egger’s test was statistically significant (*p* = 0.0487), and the funnel plot suggested asymmetry, indicating potential bias

b. 25.7% of total weight came from high-risk trials. Given the nontrivial contribution of high-risk studies we downgraded the evidence by one level

c. For SWD, length of hospital stay, and number of dressing changes, we could not assess publication bias using funnel plots or Egger’s test because each outcome included fewer than 10 studies

d. Most of the weight (78.4%) is from high-risk trials

e. Almost half (47.7%) of the total weight was contributed by trials at high risk of bias. Sensitivity analysis excluding these studies resulted in a meaningful change in the pooled effect estimate, indicating a substantial risk that bias influenced the overall result

f. Although heterogeneity was extreme (I^2^ = 99.5%, *p* < 0.00001), this was largely attributable to clear, prespecified clinical subgroup differences (mixed surgery versus knee arthroplasty). The direction of effect was consistent across subgroups, and removing any single subgroup did not reverse the overall conclusion. Given that the heterogeneity is explainable and does not undermine the consistency of the intervention’s benefit, the certainty was downgraded by one level rather than two

## Discussion

This systematic review and meta-analysis aimed to investigate whether NPWT was effective in preventing SSIs in orthopedic and trauma surgery, along with secondary results, which included SWD, length of hospital stay, and number of dressing changes. Through synthesizing data from RCTs, this review adhered to PRISMA and utilized rigorous methodologies, i.e., RoB 2 tool and GRADE assessment, to ensure the quality and reliability of the gathered evidence. Our study addresses several limitations of prior meta-analyses, which either included retrospective studies or did not capture the most recent evidence [61, 62]. By focusing exclusively on randomized controlled trials, we synthesized 18 high-quality RCTs, including new trials published, such as the first pediatric scoliosis iNPWT trial by Pérez-Acevedo et al. [60]. We also prespecified subgroup analyses for joint arthroplasty and trauma surgeries to enable context-specific comparisons and included secondary outcomes, such as wound dehiscence, length of hospital stay, and number of dressing changes, which have not been collectively analyzed in earlier reviews. The analysis of 18 RCTs involving 4585 patients demonstrated that NPWT significantly reduced the odds of SSIs by 36% (OR 0.64, 95% CI 0.50–0.83) and SWD by 61% (OR 0.39, 95% CI 0.23–0.65) compared with conventional dressings. Subgroup analyses revealed consistent benefits across joint arthroplasty (OR 0.44) and trauma surgeries (OR 0.72), with minimal heterogeneity. Additionally, NPWT was associated with a mean reduction in hospital stay of 0.87 days (95% CI −1.36 to −0.38) and fewer dressing changes.

The findings of this study were consistent with those of previous meta‐analyses. Several studies have demonstrated that NPWT significantly reduces the incidence of SSI in orthopedic surgeries. Song Yuan et al. found that NPWT lowered SSI rates across arthroplasty, fracture, and spinal surgery procedures, with a more prominent effect observed in retrospective studies compared with RCTs [62]. Similarly, Huan Liu et al. reported a significant reduction in deep SSIs, particularly in trauma surgeries, though joint and spine surgeries did not show statistically significant differences [62]. NPWT was also found to reduce the recurrence rate of infection post-discharge and reduce reoperation rates in orthopedic patients [63]. Additionally, NPWT significantly lowered the rates of both deep and superficial SSIs, as well as SWD, as reported by Cong Wang et al. [64]

Beyond infection control, numerous studies have highlighted several other benefits of NPWT. Nikhil Ailaney et al. reported a decreased length of hospital stay and reoperation rate, although NPWT was associated with a higher risk of noninfectious complications such as wound blistering [65]. Similarly, Xi Liu et al. found that NPWT significantly reduced wound healing time, length of hospital stay, and amputation rate [61]. However, a study by Grant-Freemantle et al. [66] did not find a significant difference in hospital stay duration. Furthermore, NPWT was particularly effective in reducing deep infection rates when using unsealed dressings, but this effect was insignificant when sealed dressings were applied [66].

Though NPWT offers clear advantages in infection prevention and wound healing, its impact on other complications and costs remains a subject of debate. Some studies have indicated increased medical expenses linked to its use [67], while others stress the need for further research to validate its long-term effectiveness [68, 69]. NPWT should not be viewed as a one-size-fits-all solution, as its benefits appear most meaningful in high-risk patients rather than routine, low-risk cases. Overuse in low-risk populations could lead to unnecessary costs and device-related complications without offering substantial clinical advantage. A balanced, selective approach is therefore needed to ensure that NPWT use remains both clinically meaningful and cost-conscious [67–69]. Regardless of these considerations, the collective evidence supports the beneficial role of NPWT in orthopedic surgeries.

Several of the included RCTs reported mean BMI values in the overweight or obese range (BMI 26.0–36.5), indicating that NPWT was tested in populations with elevated risk for wound complications. However, none of these studies provided outcome stratification by BMI or performed subgroup analyses. Therefore, we were unable to assess whether NPWT offers greater benefit in high-BMI subgroups. This represents an important area for future research to better define cost-effective, targeted use of NPWT.

Although this meta-analysis was carefully conducted, some limitations should be considered. The variation in patient populations, surgical procedures, and NPWT application protocols across the included studies introduces potential heterogeneity that could impact the findings. Additionally, the mode of attachment, material, and strength of NPWT likely vary from one device to another and could introduce bias that should be acknowledged. And despite utilizing statistical methods to account for these effects, residual confounding is still possible. The variation in follow-up durations also makes assessing long-term complications more difficult, primarily since most studies focused on short-term postoperative outcomes. Subgroup analysis for spine surgery could not be performed due to the presence of only one eligible study addressing this specific intervention. The financial side of NPWT has not been extensively studied, leaving us uncertain regarding its cost-effectiveness, especially in resource-limited settings. Many included RCTs enrolled high-risk patients but did not report or stratify outcomes by key comorbidities (e.g., BMI categories, diabetes), preventing any assessment of effect modification by baseline risk. Consequently, our pooled estimates may conceal clinically meaningful heterogeneity in NPWT benefit and limit patient-level applicability; future trials and IPD meta-analyses should prespecify comorbidity-adjusted and subgroup analyses to identify who benefits most and to guide cost-effective, targeted use. There were no data available to stratify outcomes based on patients’ characteristics and comorbidities, limiting our ability to highlight benefits in patients who are more comorbid. It should be noted that we included and analyzed both deep and superficial SSI, and as such, the ORs calculated represent both infection categories. One important limitation of our review is the potential for publication bias arising from our decision not to search grey literature, nonEnglish studies, or trial registries for unpublished RCTs. By focusing solely on peer-reviewed, English-language publications, we may have missed eligible trials with null or negative findings. Future updates should consider including these additional sources to provide a more comprehensive assessment. Finally, most of the included studies were conducted in high-income countries, which may limit the generalizability of the findings to regions with different healthcare infrastructures and patient demographics.

## Conclusions

NPWT appears to offer a significant clinical benefit in reducing the incidence of SSIs in orthopedic and trauma surgery. Secondary analyses also demonstrated benefits for surgical wound dehiscence, length of hospital stay, and number of dressing changes. However, the certainty of evidence is moderate, and these findings should be interpreted with caution. Further well-designed, multicenter RCTs are warranted to confirm these benefits, assess long-term outcomes, and evaluate cost-effectiveness.

## Supplementary Information

Additional file1.

## Data Availability

All datasets generated or analyzed during the current study are presented in the main paper and the supplementary files.

## Abbreviations

SSI: Surgical site infection
NPWT: Negative pressure wound therapy
iNPWT: Incisional negative pressure wound therapy
S. aureus: Staphylococcus aureus
NICE: National institute for health and care excellence
WHO: World Health Organization
SWD: Surgical wound dehiscence
RCT: Randomized controlled trial
PRISMA: Preferred reporting items for systematic reviews and meta-analyses
PROSPERO: International prospective register of systematic reviews
MeSH: Medical subject headings
RoB 2: Risk of bias 2
OR: Odds ratio
CI: Confidence interval
MD: Mean difference
SD: Standard deviation
GRADE: Grading of recommendations, assessment, development, and evaluations
BMI: Body mass index
CD: Conventional dressing
p: P-value
NA: Not applicable
