# Supplementary material for: Negative pressure wound therapy for the prevention of surgical site infections in orthopedic and trauma surgery: a systematic review and meta-analysis of RCTs
Source: J Orthop Traumatol. 2025 Dec 16;26:75. doi: 10.1186/s10195-025-00889-0 (PMC12708484; doi:10.1186/s10195-025-00889-0)
Supplement: Supplementary file 1 — Additional file1. [file 10195_2025_889_MOESM1_ESM.docx]

**Appendix 1: Search History**

**1.PubMed**

| **Search number** | **Query** | **Results** |
| --- | --- | --- |
| 1 | "Orthopedics"[Mesh] | 25,354 |
| 2 | orthopedic surgery[Title/Abstract] | 11,637 |
| 3 | Orthopedics[Title/Abstract] | 15,242 |
| 4 | (((orthopedic trauma surgery[Title/Abstract]) OR (orthopedic surgery[Title/Abstract])) OR (spinal surgery[Title/Abstract])) OR (arthroplasty[Title/Abstract]) | 118,968 |
| 5 | ((("Orthopedics"[Mesh]) OR (orthopedic surgery[Title/Abstract])) OR (Orthopedics[Title/Abstract])) OR ((((orthopedic trauma surgery[Title/Abstract]) OR (orthopedic surgery[Title/Abstract])) OR (spinal surgery[Title/Abstract])) OR (arthroplasty[Title/Abstract])) | 151,105 |
| 6 | "Surgical Wound Infection"[Mesh] | 42,821 |
| 7 | (((((((((((((((Surgical Wound Infection[Title/Abstract]) OR (Infections, Surgical Wound[Title/Abstract])) OR (Surgical Wound Infections[Title/Abstract])) OR (Wound Infections, Surgical[Title/Abstract])) OR (Infection, Surgical Wound[Title/Abstract])) OR (Surgical Site Infection[Title/Abstract])) OR (Infection, Surgical Site[Title/Abstract])) OR (Infections, Surgical Site[Title/Abstract])) OR (Surgical Site Infections[Title/Abstract])) OR (Wound Infection, Postoperative[Title/Abstract])) OR (Wound Infection, Surgical[Title/Abstract])) OR (Infection, Postoperative Wound[Title/Abstract])) OR (Infections, Postoperative Wound[Title/Abstract])) OR (Postoperative Wound Infections[Title/Abstract])) OR (Wound Infections, Postoperative[Title/Abstract])) OR (Postoperative Wound Infection[Title/Abstract]) | 23,755 |
| 8 | ("Surgical Wound Infection"[Mesh]) OR ((((((((((((((((Surgical Wound Infection[Title/Abstract]) OR (Infections, Surgical Wound[Title/Abstract])) OR (Surgical Wound Infections[Title/Abstract])) OR (Wound Infections, Surgical[Title/Abstract])) OR (Infection, Surgical Wound[Title/Abstract])) OR (Surgical Site Infection[Title/Abstract])) OR (Infection, Surgical Site[Title/Abstract])) OR (Infections, Surgical Site[Title/Abstract])) OR (Surgical Site Infections[Title/Abstract])) OR (Wound Infection, Postoperative[Title/Abstract])) OR (Wound Infection, Surgical[Title/Abstract])) OR (Infection, Postoperative Wound[Title/Abstract])) OR (Infections, Postoperative Wound[Title/Abstract])) OR (Postoperative Wound Infections[Title/Abstract])) OR (Wound Infections, Postoperative[Title/Abstract])) OR (Postoperative Wound Infection[Title/Abstract])) | 54,021 |
| 9 | "Negative-Pressure Wound Therapy"[Mesh] | 4,420 |
| 10 | ((((((((((((((((((((((Negative-Pressure Wound Therapies[Title/Abstract]) OR (Negative Pressure Wound Therapy[Title/Abstract])) OR (Therapies, Negative-Pressure Wound[Title/Abstract])) OR (Therapy, Negative-Pressure Wound[Title/Abstract])) OR (Wound Therapies, Negative-Pressure[Title/Abstract])) OR (Wound Therapy, Negative-Pressure[Title/Abstract])) OR (Topical Negative-Pressure Therapy[Title/Abstract])) OR (Negative-Pressure Therapies, Topical[Title/Abstract])) OR (Negative-Pressure Therapy, Topical[Title/Abstract])) OR (Therapies, Topical Negative-Pressure[Title/Abstract])) OR (Therapy, Topical Negative-Pressure[Title/Abstract])) OR (Topical Negative-Pressure Therapies[Title/Abstract])) OR (Topical Negative Pressure Therapy[Title/Abstract])) OR (Vacuum-Assisted Closure[Title/Abstract])) OR (Closures, Vacuum-Assisted[Title/Abstract])) OR (Closure, Vacuum-Assisted[Title/Abstract])) OR (Vacuum Assisted Closure[Title/Abstract])) OR (Vacuum-Assisted Closures[Title/Abstract])) OR (Negative-Pressure Dressings[Title/Abstract])) OR (Dressing, Negative-Pressure[Title/Abstract])) OR (Dressings, Negative-Pressure[Title/Abstract])) OR (Negative-Pressure Dressing[Title/Abstract])) OR (Negative Pressure Dressings[Title/Abstract]) | 5,891 |
| 11 | ("Negative-Pressure Wound Therapy"[Mesh]) OR (((((((((((((((((((((((Negative-Pressure Wound Therapies[Title/Abstract]) OR (Negative Pressure Wound Therapy[Title/Abstract])) OR (Therapies, Negative-Pressure Wound[Title/Abstract])) OR (Therapy, Negative-Pressure Wound[Title/Abstract])) OR (Wound Therapies, Negative-Pressure[Title/Abstract])) OR (Wound Therapy, Negative-Pressure[Title/Abstract])) OR (Topical Negative-Pressure Therapy[Title/Abstract])) OR (Negative-Pressure Therapies, Topical[Title/Abstract])) OR (Negative-Pressure Therapy, Topical[Title/Abstract])) OR (Therapies, Topical Negative-Pressure[Title/Abstract])) OR (Therapy, Topical Negative-Pressure[Title/Abstract])) OR (Topical Negative-Pressure Therapies[Title/Abstract])) OR (Topical Negative Pressure Therapy[Title/Abstract])) OR (Vacuum-Assisted Closure[Title/Abstract])) OR (Closures, Vacuum-Assisted[Title/Abstract])) OR (Closure, Vacuum-Assisted[Title/Abstract])) OR (Vacuum Assisted Closure[Title/Abstract])) OR (Vacuum-Assisted Closures[Title/Abstract])) OR (Negative-Pressure Dressings[Title/Abstract])) OR (Dressing, Negative-Pressure[Title/Abstract])) OR (Dressings, Negative-Pressure[Title/Abstract])) OR (Negative-Pressure Dressing[Title/Abstract])) OR (Negative Pressure Dressings[Title/Abstract])) | 7,162 |
| 12 | ((((("Orthopedics"[Mesh]) OR (orthopedic surgery[Title/Abstract])) OR (Orthopedics[Title/Abstract])) OR ((((orthopedic trauma surgery[Title/Abstract]) OR (orthopedic surgery[Title/Abstract])) OR (spinal surgery[Title/Abstract])) OR (arthroplasty[Title/Abstract]))) AND (("Surgical Wound Infection"[Mesh]) OR ((((((((((((((((Surgical Wound Infection[Title/Abstract]) OR (Infections, Surgical Wound[Title/Abstract])) OR (Surgical Wound Infections[Title/Abstract])) OR (Wound Infections, Surgical[Title/Abstract])) OR (Infection, Surgical Wound[Title/Abstract])) OR (Surgical Site Infection[Title/Abstract])) OR (Infection, Surgical Site[Title/Abstract])) OR (Infections, Surgical Site[Title/Abstract])) OR (Surgical Site Infections[Title/Abstract])) OR (Wound Infection, Postoperative[Title/Abstract])) OR (Wound Infection, Surgical[Title/Abstract])) OR (Infection, Postoperative Wound[Title/Abstract])) OR (Infections, Postoperative Wound[Title/Abstract])) OR (Postoperative Wound Infections[Title/Abstract])) OR (Wound Infections, Postoperative[Title/Abstract])) OR (Postoperative Wound Infection[Title/Abstract])))) AND (("Negative-Pressure Wound Therapy"[Mesh]) OR (((((((((((((((((((((((Negative-Pressure Wound Therapies[Title/Abstract]) OR (Negative Pressure Wound Therapy[Title/Abstract])) OR (Therapies, Negative-Pressure Wound[Title/Abstract])) OR (Therapy, Negative-Pressure Wound[Title/Abstract])) OR (Wound Therapies, Negative-Pressure[Title/Abstract])) OR (Wound Therapy, Negative-Pressure[Title/Abstract])) OR (Topical Negative-Pressure Therapy[Title/Abstract])) OR (Negative-Pressure Therapies, Topical[Title/Abstract])) OR (Negative-Pressure Therapy, Topical[Title/Abstract])) OR (Therapies, Topical Negative-Pressure[Title/Abstract])) OR (Therapy, Topical Negative-Pressure[Title/Abstract])) OR (Topical Negative-Pressure Therapies[Title/Abstract])) OR (Topical Negative Pressure Therapy[Title/Abstract])) OR (Vacuum-Assisted Closure[Title/Abstract])) OR (Closures, Vacuum-Assisted[Title/Abstract])) OR (Closure, Vacuum-Assisted[Title/Abstract])) OR (Vacuum Assisted Closure[Title/Abstract])) OR (Vacuum-Assisted Closures[Title/Abstract])) OR (Negative-Pressure Dressings[Title/Abstract])) OR (Dressing, Negative-Pressure[Title/Abstract])) OR (Dressings, Negative-Pressure[Title/Abstract])) OR (Negative-Pressure Dressing[Title/Abstract])) OR (Negative Pressure Dressings[Title/Abstract]))) | 97 |

**2. Cochrane library**

| **Search number** | **Query** | **Results** |
| --- | --- | --- |
| 1 | MeSH descriptor: [Orthopedics] explode all trees | *580* |
| 2 | (orthopedic trauma surgery):ti,ab,kw OR (orthopedic surgery):ti,ab,kw OR (spinal surgery):ti,ab,kw OR (arthroplasty):ti,ab,kw | 40272 |
| 3 | MeSH descriptor: [Negative-Pressure Wound Therapy] explode all trees | *427* |
| 4 | (Negative-Pressure Wound Therapies):ti,ab,kw OR (Negative Pressure Wound Therapy):ti,ab,kw OR (Negative-Pressure Wound Therapy):ti,ab,kw OR (Therapies, Negative-Pressure Wound):ti,ab,kw OR (Therapy, Negative-Pressure Wound):ti,ab,kw | 1146 |
| 5 | (Wound Therapies, Negative-Pressure):ti,ab,kw OR (Wound Therapy, Negative-Pressure):ti,ab,kw OR (Topical Negative-Pressure Therapy):ti,ab,kw OR (Negative-Pressure Therapies, Topical):ti,ab,kw AND (Negative-Pressure Therapy, Topical):ti,ab,kw | 1095 |
| 6 | (Therapies, Topical Negative-Pressure):ti,ab,kw OR (Therapy, Topical Negative-Pressure):ti,ab,kw OR (Topical Negative-Pressure Therapies):ti,ab,kw OR (Topical Negative Pressure Therapy):ti,ab,kw OR (Vacuum-Assisted Closure):ti,ab,kw | 751 |
| 7 | (Closures, Vacuum-Assisted):ti,ab,kw OR (Closure, Vacuum-Assisted):ti,ab,kw OR (Vacuum Assisted Closure):ti,ab,kw OR (Vacuum-Assisted Closures):ti,ab,kw OR (Negative-Pressure Dressings):ti,ab,kw | 871 |
| 8 | (Dressing, Negative-Pressure):ti,ab,kw OR (Dressings, Negative-Pressure):ti,ab,kw OR (Negative-Pressure Dressing):ti,ab,kw OR (Negative Pressure Dressings):ti,ab,kw | 788 |
| 9 | MeSH descriptor: [Surgical Wound Infection] explode all trees | *4539* |
| 10 | (Surgical Wound Infection):ti,ab,kw OR (Infections, Surgical Wound):ti,ab,kw OR (Surgical Wound Infections):ti,ab,kw OR (Wound Infections, Surgical):ti,ab,kw OR (Infection, Surgical Wound):ti,ab,kw | 9946 |
| 11 | (Surgical Site Infection):ti,ab,kw OR (Infection, Surgical Site):ti,ab,kw OR (Infections, Surgical Site):ti,ab,kw OR (Surgical Site Infections):ti,ab,kw OR (Wound Infection, Postoperative):ti,ab,kw | 11376 |
| 12 | (Wound Infection, Surgical):ti,ab,kw OR (Infection, Postoperative Wound):ti,ab,kw OR (Infections, Postoperative Wound):ti,ab,kw OR (Postoperative Wound Infections):ti,ab,kw OR (Wound Infections, Postoperative):ti,ab,kw | 11673 |
| 13 | #1 or #2 | 40531 |
| 14 | #3 or #4 or #5 or #6 or #7 or #8 | 1471 |
| 15 | #9 or #10 or #11 or #12 | 14287 |
| 16 | #13 and #14 and #15 | 55 |

**3. Scopus**

| **Search Number** | **Query** | **Results** |
| --- | --- | --- |
| 1 | (TITLE-ABS-KEY(Orthopedics) OR TITLE-ABS-KEY(orthopedic trauma surgery) OR TITLE-ABS-KEY(orthopedic surgery) OR TITLE-ABS-KEY(spinal surgery) OR TITLE-ABS-KEY(arthroplasty)) | 489,223 |
| 2 | ( TITLE-ABS-KEY ( negative-pressure AND wound AND therapy ) OR TITLE-ABS-KEY ( negative-pressureAND wound AND therapies ) OR TITLE-ABS-KEY ( negative AND pressure AND wound AND therapy ) OR TITLE-ABS-KEY ( therapies, AND negative-pressure AND wound ) OR TITLE-ABS-KEY ( therapy, AND negative-pressure AND wound ) OR TITLE-ABS-KEY ( wound AND therapies, AND negative-pressure ) OR TITLE-ABS-KEY ( wound AND therapy, AND negative-pressure ) OR TITLE-ABS-KEY ( topical AND negative-pressure AND therapy ) OR TITLE-ABS-KEY ( negative-pressure AND therapies, AND topical ) OR TITLE-ABS-KEY ( negative-pressure AND therapy, AND topical ) OR TITLE-ABS-KEY ( therapies, AND topical AND negative-pressure ) OR TITLE-ABS-KEY ( therapy, AND topical AND negative-pressure ) OR TITLE-ABS-KEY ( topical AND negative-pressure AND therapies ) OR TITLE-ABS-KEY ( topical AND negative AND pressure AND therapy ) OR TITLE-ABS-KEY ( vacuum-assisted AND closure ) OR TITLE-ABS-KEY ( closures, AND vacuum-assisted ) OR TITLE-ABS-KEY ( closure, AND vacuum-assisted ) OR TITLE-ABS-KEY ( vacuum AND assisted AND closure ) OR TITLE-ABS-KEY ( vacuum-assisted AND closures ) OR TITLE-ABS-KEY ( negative-pressure AND dressings ) OR TITLE-ABS-KEY ( dressing, AND negative-pressure ) OR TITLE-ABS-KEY ( dressings, AND negative-pressure ) OR TITLE-ABS-KEY ( negative-pressureAND dressing ) OR TITLE-ABS-KEY ( negative AND pressure AND dressings ) ) | 13,117 |
| 3 | (TITLE-ABS-KEY(Surgical Wound Infection) OR TITLE-ABS-KEY(Infections, Surgical Wound) OR TITLE-ABS-KEY(Surgical Wound Infections) OR TITLE-ABS-KEY(Wound Infections, Surgical) OR TITLE-ABS-KEY(Infection, Surgical Wound) OR TITLE-ABS-KEY(Surgical Site Infection) OR TITLE-ABS-KEY(Infection, Surgical Site) OR TITLE-ABS-KEY(Infections, Surgical Site) OR TITLE-ABS-KEY(Surgical Site Infections) OR TITLE-ABS-KEY(Wound Infection, Postoperative) OR TITLE-ABS-KEY(Wound Infection, Surgical) OR TITLE-ABS-KEY(Infection, Postoperative Wound) OR TITLE-ABS-KEY(Infections, Postoperative Wound) OR TITLE-ABS-KEY(Postoperative Wound Infections) OR TITLE-ABS-KEY(Wound Infections, Postoperative) OR TITLE-ABS-KEY(Postoperative Wound Infection)) | 138,254 |
| 4 | #1 AND # 2 AND #3 | 615 |

**4.Web of science**

| **Search number** | **Query** | **Results** |
| --- | --- | --- |
| 1 | ((((TS=(Orthopedics)) OR TS=(orthopedic trauma surgery)) OR TS=(orthopedic surgery)) OR TS=(spinal surgery)) OR TS=(arthroplasty) | 223,755 |
| 2 | ((((((((((((((((((((((TS=(Negative-Pressure Wound Therapy) OR TS=(Negative-Pressure Wound Therapies) OR TS=(Negative Pressure Wound Therapy)) OR TS=(Therapies, Negative-Pressure Wound)) OR TS=(Therapy, Negative-Pressure Wound)) OR TS=(Wound Therapies, Negative-Pressure)) OR TS=(Wound Therapy, Negative-Pressure)) OR TS=(Topical Negative-Pressure Therapy)) OR TS=(Negative-Pressure Therapies, Topical)) OR TS=(Negative-Pressure Therapy, Topical)) OR TS=(Therapies, Topical Negative-Pressure)) OR TS=(Therapy, Topical Negative-Pressure)) OR TS=(Topical Negative-Pressure Therapies)) OR TS=(Topical Negative Pressure Therapy)) OR TS=(Vacuum-Assisted Closure)) OR TS=(Closures, Vacuum-Assisted)) OR TS=(Closure, Vacuum-Assisted)) OR TS=(Vacuum Assisted Closure)) OR TS=(Vacuum-Assisted Closures)) OR TS=(Negative-Pressure Dressings)) OR TS=(Dressing, Negative-Pressure)) OR TS=(Dressings, Negative-Pressure)) OR TS=(Negative-Pressure Dressing)) OR TS=(Negative Pressure Dressings)) | 8,055 |
| 3 | (((((((((((((((TS=(Surgical Wound Infection)) OR TS=(Infections, Surgical Wound)) OR TS=(Surgical Wound Infections)) OR TS=(Wound Infections, Surgical)) OR TS=(Infection, Surgical Wound)) OR TS=(Surgical Site Infection)) OR TS=(Infection, Surgical Site)) OR TS=(Infections, Surgical Site)) OR TS=(Surgical Site Infections)) OR TS=(Wound Infection, Postoperative)) OR TS=(Wound Infection, Surgical)) OR TS=(Infection, Postoperative Wound)) OR TS=(Infections, Postoperative Wound)) OR TS=(Postoperative Wound Infections)) OR TS=(Wound Infections, Postoperative)) OR TS=(Postoperative Wound Infection) | 59,640 |
| 4 | #1 AND #2 AND #3 | 215 |

**Appendix 2: Risk of bias assessment**

**Appendix 2.1: Risk of bias assessment table**

| Study ID | D1: Randomisation process | Reason | D2: Deviations from intended interventions | Reason | D3: Missing outcome data | Reason | D4: Measurement of the outcome | Reason | D5: Selection of the reported result | Reason | ****Overall RoB**** |
| --- | --- | --- | --- | --- | --- | --- | --- | --- | --- | --- | --- |
| ****Virani et al.**** | Low risk | - | Low risk | - | Low risk | - | Low risk | - | Low risk | - | **Low risk** |
| ****Cooper et al.**** | Low risk | - | Some concerns | No blinding of patients or surgeons | Low risk | - | Low risk | - | Low risk | - | **Some concerns** |
| ****Newman et al.**** | Low risk | - | Low risk | - | Some concerns | Unclear handling of exclusions and ITT adherence | Low risk | - | Low risk | - | **Some concerns** |
| ****Karlakki et al.**** | Low risk | - | Some concerns | Non-blinded trial | Low risk | - | High risk | Non-blinded outcome assessment; reliance on phone checks for most complications | Low risk | - | **High risk** |
| ****Costa et al.**** | Low risk | - | Low risk | - | Low risk | - | Low risk | - | Low risk | - | **Low risk** |
| ****Pérez-Acevedo et al.**** | Low risk | - | Low risk | - | High risk | 21.2% dropout rate (14/66 patients) | Low risk | - | Low risk | - | **High risk** |
| ****Manoharran et al.**** | Low risk | - | High risk | Open-label trial; surgeons informed of allocation before surgery | Low risk | - | Some concerns | Final wound assessment by unblinded surgeons/nurses; no independent assessors; patients unblinded for wound diaries | Low risk | - | **High risk** |
| ****Masters et al.**** | Low risk | - | Low risk | - | Low risk | - | Low risk | - | Low risk | - | **Low risk** |
| ****Cai et al.**** | Low risk | - | Low risk | - | High risk | 12 patients lost to follow-up (5 VSD, 7 conventional) | Low risk | - | Low risk | - | **High risk** |
| ****Giannini et al.**** | Low risk | - | Low risk | - | Low risk | - | Low risk | - | Low risk | - | **Low risk** |
| ****Keeny et al.**** | Low risk | - | Low risk | - | Some concerns | 17.8% attrition; exclusions documented but could affect results | Low risk | - | Low risk | - | **Some concerns** |
| ****Gillespie et al.**** | Low risk | - | High risk | No blinding; two NPWT patients received standard dressings; co-intervention (early dressing removal) in NPWT group could bias results | Some concerns | >10% attrition by 6 weeks in both groups | Low risk | - | Low risk | - | **High risk** |
| ****Arti et al.**** | Low risk | - | Low risk | - | Low risk | - | Low risk | - | Low risk | - | **Low risk** |
| ****Canton et al.**** | Low risk | - | Low risk | - | Low risk | - | Low risk | - | Low risk | - | **Low risk** |
| ****Higuera-Rueda et al.**** | Low risk | - | Some concerns | Open-label design post-surgery; no blinding of participants/staff | Low risk | - | Low risk | - | Low risk | - | **Some concerns** |
| ****Stannard et al.**** | Low risk | - | Low risk | - | Low risk | - | Low risk | - | Some concerns | Industry funding from NPWT manufacturer introduces potential bias | **Some concerns** |
| ****Crist et al.**** | Low risk | - | Some concerns | No blinding of patients/staff; possible variation in NPWT application; no log of therapy interruptions | Low risk | - | Low risk | - | Low risk | - | **Some concerns** |
| ****Costa et al.**** | Low risk | - | Low risk | - | Low risk | - | Low risk | - | Low risk | - | **Low risk** |

**Appendix 2.2: The overall risk of bias for all included studies.**


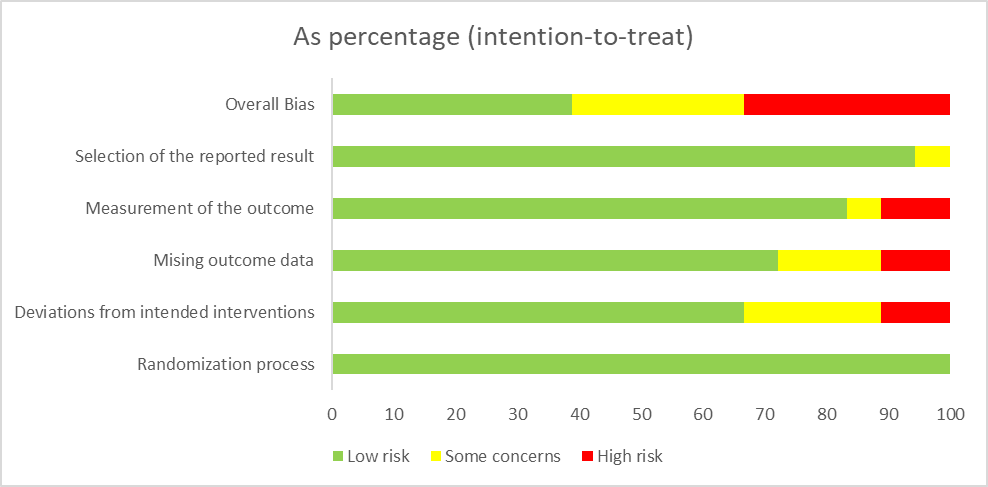


# **Appendix 3: Handling Missing Data and Data Transformation**

## ***1. Estimation of Missing Standard Deviations (SDs)***

### A. From p-values of t-tests

When a p-value for the comparison of means was reported without SD, the SD was calculated as:

SD = Mean difference / ( t / √n )

where t is the t-statistic corresponding to the reported p-value and degrees of freedom, and n is the sample size.

### B. From Confidence Intervals (CI)

When 95% confidence intervals (CI) for means or mean differences were reported without SD, the SD was estimated by:

SD = (Upper limit − Lower limit) / ( 2 × t_df,0.975 × √n )

where t_df,0.975 is the critical t-value for the given degrees of freedom.

## ***2. Conversion of Median and Interquartile Range (IQR) to Mean and SD***

When studies reported medians and IQR instead of means and SDs, these were converted using Wan et al.'s method (2014) as follows:

Mean ≈ (Q1 + Q2 + Q3) / 3

where Q1 and Q3 are the 25th and 75th percentiles (lower and upper quartiles), and Q2 is the median.

SD ≈ (Q3 − Q1) / 1.35

This formula assumes an approximately normal distribution.

## Notes:

• Degrees of freedom (df) were calculated as n − 1.

• t-values were obtained from standard t-distribution tables.

• When appropriate, pooled SDs were calculated assuming equal variances.

## References

Wan X, Wang W, Liu J, Tong T. Estimating the sample mean and standard deviation from the sample size, median, range and/or interquartile range. BMC Med Res Methodol. 2014;14:135.

Cochrane Handbook for Systematic Reviews of Interventions Version 6.4 (2023). Chapter 6.5.2.3: Dealing with missing standard deviations.
